# Clinical, radiographic and histopathological analysis of bone-forming tumors of the oral and maxillofacial region: A 53-year retrospective study

**DOI:** 10.4317/medoral.27793

**Published:** 2026-03-07

**Authors:** Maria Carolina Magalhães de Carvalho, Débora Frota Colares, Hannah Gil de Farias Morais, Amanda Katarinny Goes Gonzaga, Antônio de Lisboa Lopes Costa, Pedro Paulo de Andrade Santos, Lélia Batista de Souza

**Affiliations:** 1 Department of Oral Pathology, Federal University of Rio Grande do Norte, Natal, RN, Brazil; 2 Multicampi School of Medical Sciences, Federal University of Rio Grande do Norte, Caicó, RN, Brazil; 3 Department of Morphology, Biosciences Center, Federal University of Rio Grande do Norte, Natal, RN, Brazil

## Abstract

**Background:**

Non-odontogenic bone tumors of the jawbones constitute a heterogeneous group of tumors that may arise in various regions of the maxillofacial complex and present as chondroid or bone-forming tumors (BFTs). Therefore, this 53-year retrospective study aimed to evaluate the clinical, radiographic, and histopathological characteristics of BFTs (osteoma-OT, osteoblastoma-OB and osteosarcoma-OS) diagnosed at a single oral pathology referral center in Brazil.

**Material and Methods:**

Gender, age, symptoms, clinical diagnosis, lesion duration, anatomical site, size of the lesion, and radiographic characteristics were collected from all cases previously diagnosed as BFTs, and all histological sections were reviewed.

**Results:**

Among 19.596 cases diagnosed at the service during the study period, 74 (0.37%) were identified as OTs, OBs or OSs, with OTs being the most common (55.4%). There was a predominance of female patients (70.2%), and the mandible was the most affected gnathic bone (84.7%). Radiographically, OTs typically presented as well-delimited, radiopaque lesions (68.4%), whereas OBs (68.4%) and OSs (75.0%) frequently exhibited mixed radiolucent patterns. Histopathologically, OTs were predominantly classified as compact, and OBs were commonly typical. Osteosarcomas were frequently of osteoblastic subtype.

**Conclusions:**

Awareness of clinical, radiographic and histopathological characteristics is essential for improving recognition of the distinct profiles of BFTs.

## Introduction

Non-odontogenic bone neoplasms encompass a range of benign and malignant tumors that may develop in various regions of the maxillomandibular complex. Among these, bone-forming tumors (BFTs) of the gnathic bones are notable for overlapping clinical and radiographic features, yet they exhibit distinct histopathological profiles (1 - 4). These entities include osteoma (OT), osteoblastoma (OB), and osteosarcoma (OS). Their etiology remains unclear, although both genetic factors and local risk determinants have been implicated (1 , 3 - 10).

Due to the rarity of these neoplasms and the occasional overlap of clinicopathological features, challenges arise in clinical practice, which may result an inappropriate management and potential diagnostic delays (3 , 4). This scenario stems primarily from the low frequency of BFTs in gnathic sites and, more importantly, from the limited knowledge regarding the clinical, radiographic, and morphological profiles of these conditions (3 - 4 , 7).

Given the scarcity of retrospective studies investigating the clinicopathological profile of BFTs of the gnathic bones, research integrating this information is essential to improve diagnostic accuracy for clinicians and oral pathologists. Accordingly, this study aimed to analyze the occurrence of BFTs at a referral Oral Pathology Service in Brazil, to characterize their clinical, radiographic, and histopathological features, and to compare these findings with current literature.

## Material and Methods

Study design

The Ethics Committee of the Federal University of Rio Grande do Norte (UFRN) approved the present study (Approval No. 6.712.511). This retrospective cross-sectional study reviewed and analyzed the clinical, radiographic, and histopathological characteristics of cases of BFTs diagnosed from January 1970 to December 2023 at the Oral Pathology Service of the Dentistry Department of UFRN (Natal, RN, Brazil), a Brazilian oral and maxillofacial pathology referral center.

All lesions were diagnosed based on the correlation of clinical, radiographic, and histopathological characteristics. Bone-forming tumors of the jaws were categorized according to the World Health Organization (WHO) classification of Head and Neck Tumors as osteoma (OT), osteoblastoma (OB), or osteosarcoma (OS) (1 , 3 , 4 , 11 - 13).

Cases with sufficient clinical and radiographic information -either in the form of available radiographic exams or as radiographic details provided by the clinician- were included to allow accurate diagnosis. Cases in which the available data were insufficient to establish the final diagnosis were excluded. Cases with inadequate formalin-fixed paraffin-embedded material to review the histopathological features and cases diagnosed as other intraosseous lesions during the morphological analysis were excluded.

Clinical-radiographic analysis

Data regarding gender, age, skin color, clinical presentation, symptoms, anatomical location, lesion size and duration, and clinical diagnosis were obtained from the biopsy records. Cases with available radiographic data (either as imaging examinations or as radiographic details reported by the referring clinicians in the biopsy records) were analyzed for radiodensity (radiopaque, radiolucent, or mixed), lesion borders (well-defined or poorly defined), and presence of a halo. Additionally, specific features were assessed for OTs, such as the presence of a central nidus, and OSs (periodontal ligament thickening, root resorption and periosteal reaction).

Histopathological analysis

For histopathological analysis, slides containing hematoxylin and eosin-stained 5-µm-thick sections were reassessed. Two examiners (MCMC and DFC), previously trained by an experienced oral pathologist (LBS), conducted the analysis. The examiners independently analyzed the slides of BFTs under an optical microscope (Nikon Eclipse-E200, Tokyo, Japan). Any discrepancies between the examiners were resolved through discussion among all three researchers to reach a consensus.

The examiners initially reassessed the histopathological features on each slide to confirm the diagnosis and, if necessary, reclassified the lesions according to the latest WHO Classification of Head and Neck Tumors (1 , 3 - 5 , 8 , 11 - 13). Briefly, lesions were classified as OTs when they presented as masses of spongy or trabecular bone with prominent Haversian channels and low cellularity (5 , 11 , 14). Cases exhibiting osteogenic tumors characterized by anastomosing trabeculae of bone and osteoid rimmed by plump osteoblasts, set within a richly vascular fibrous stroma, were diagnosed as OBs (3 , 8 , 12 , 14). Finally, cases were diagnosed as OSs when composed of atypical cells producing immature neoplastic osteoid, distributed within osteoblastic, chondroblastic, or fibroblastic matrices (13 , 15).

Subsequently, the histopathological features were analyzed in detail throughout the slide using a zigzag pattern to ensure consistency between the two examiners. Osteomas were classified according to Boffano (5) as compact or cancellous. Cases identified as osteoid osteomas (OOTs) were analyzed regarding the nidus maturation as initial, intermediate or mature, according to the method proposed by Yalcinkaya (8). Osteoblastomas were analyzed regarding their cellular density, presence of epithelioid atypical osteoblasts, multinucleated giant cells, chondroid matrix, as well as the association with aneurysmal bone cysts (8). Finally, OSs cases were categorized according to the presence of cellular atypia, degree of differentiation and stromal characteristics (3 , 4 , 13 , 15).

Statistical Analysis

Data were tabulated and analyzed using descriptive statistics using the IBM SPSS software (version 22.0; IBM Corp., USA). Pearson's chi-square test and Fisher's exact test were used to analyze the associations between categorical variables. The Kolmogorov-Smirnov test was used to determine the normality of the data. The Kruskal-Wallis test was used for associations between categorical and numerical variables. A level of significance of 5% (p &lt; 0.05) was adopted. The kappa coefficient () was calculated to assess the interobserver agreement in the histopathological analysis (0.20, slight agreement; 0.21-0.40, fair agreement; 0.41-0.60, moderate agreement; 0.61-0.80, good agreement; 0.81-1, excellent agreement).

## Results

Sampling

Among 19.596 cases of oral and maxillofacial lesions, 82 cases were diagnosed as BFTs. After the histopathological re-evaluation and exclusion specimens with inappropriate material for histological reassessment, 74 (0.37%) cases of BFTs were included in the present study. These cases were diagnosed between 1970 and 2023. A progressive increase in diagnoses over the decades was observed, with a peak during the decade from 2010 to 2019 (n=38; 51.4%), followed by a decrease after this period likely due to the COVID-19 pandemic (n=5; 6.8%). A total of 41 (55.4%) cases of OT, 17 (23%) cases of OB and 16 (21.6%) of OS were identified.

Demographic data, clinical aspects, and radiographic features

The demographic data and clinical aspects of the included cases are presented in Table 1. White patients (43.2%) were frequently affected, and female-to-male ratio was 2.36:1. The mean age of patients with BFTs was 36.72±18.66 years (range 7-79 years). Most cases presented as swellings (n=38; 51.3%) and affected the posterior mandible (n=47; 65.3%). Furthermore, seven cases did not exhibit detectable clinical alterations. Benign BFTs (OTs and OBs) were mostly asymptomatic, whereas most OS patients reported the presence of symptoms (63.6%).

Table 1Osteomas had a long disease duration at diagnosis with a maximum size of 2.5cm, whereas OB and OS cases had shorter duration and larger diameters (Table 1). Three cases of OB had sizes larger than 4cm (range 5-7cm) (17.6%). While most clinical diagnoses of OT were concordant with the final diagnoses, cases that were ultimately diagnosed as either OB or OS were clinically interpreted as other intraosseous lesions, including ossifying fibroma, central giant cell granuloma, fibrous dysplasia, and odontogenic tumors.

**Figure 1:**
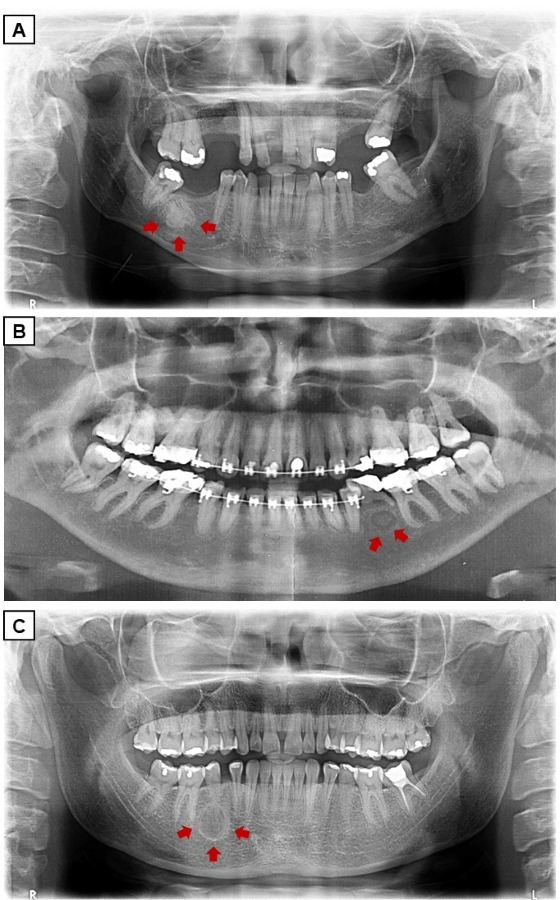
Radiographic characteristics of benign bone-forming tumors of the jaws on panoramic radiographs. (A) Osteoma exhibiting a homogeneous, well-defined radiopaque lesion, located in the edentulous region of the right mandibular body corresponding to the region of tooth 47. (B) Osteoid osteoma presenting as a well-defined radiopaque lesion, surrounded by a radiolucent halo, located in the periapical region of tooth 75 (the patient had agenesis of tooth 35). (C) Osteoblastoma showing a well-defined radiolucent lesion, surrounded by a radiopaque halo, located in the periapical region of tooth 45 in the right mandibular body.

**Figure 2:**
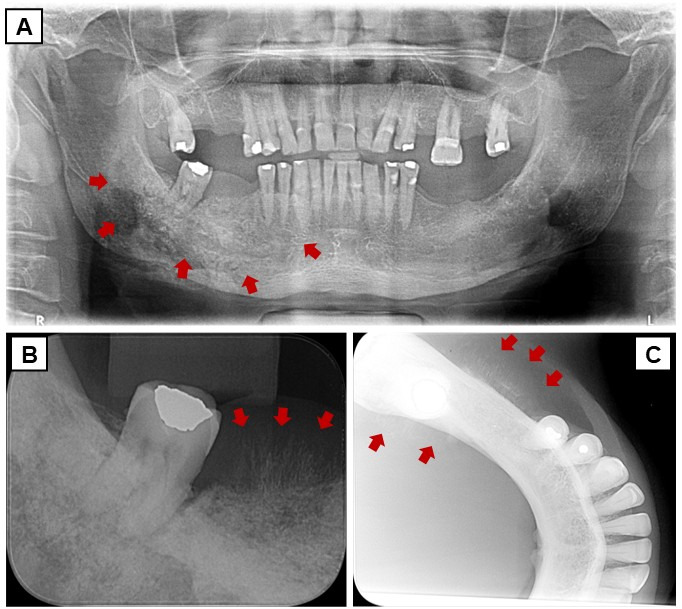
Radiographic characteristics of osteosarcomas of the jaws (panoramic, periapical, and occlusal radiographs). (A) A mixed radiolucent-radiopaque lesion with poorly defined margins affecting the right mandibular body and ramus. (B) Absence of the lamina dura and thickening of the periodontal ligament space around tooth 47. In the edentulous region, a periosteal reaction with a classic “sunburst” pattern is observed, characterized by radiopaque bone spicules arranged perpendicular to the cortical bone (red arrows). (C) Occlusal radiograph demonstrating the characteristic sunburst periosteal reaction shown in panel B (red arrows).

Radiographic characteristics are presented in Figure 1 (benign BFTs), Figure 2 (OSs) and Table 2. Osteomas were mostly radiopaque (n=21). In contrast, a high frequency of mixed radiolucent pattern was observed in OB (n=11) and OS (n=7). Lesion borders were commonly well-defined in OT (n=13) and OB (n=6), but poorly defined in OS (n=8).

1

2

Table 2

**Figure 3:**
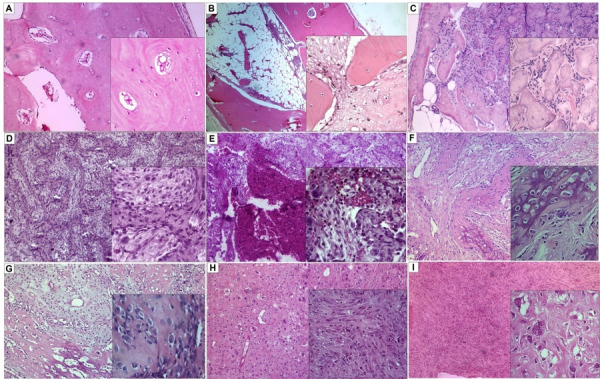
Histopathological features of bone-forming tumors of the jaws. (A) Compact osteoma showing sparse medullary spaces. The inset displays concentric lamellae associated with Haversian canal system. (B) Cancellous osteoma displaying wide medullary spaces (inset). (C) Osteoid osteoma showing nidus formation in the central region. The inset exhibits cuboidal and spindle-shaped cells producing osteoid and immature bone tissue. (D, E and F) Osteoblastoma. In D, bony trabeculae at various stages of maturation within a fibrous stroma are demonstrated. Osteoblastic rimming, large stellate and spindle-shaped osteoblasts, and multinucleated giant cells are also observed (inset). In E, a case of osteoblastoma associated with an aneurysmal bone cyst, exhibiting dilated capillaries, giant cells, and osteoblastic cells (inset). In F, a clinically aggressive osteoblastoma presenting osteoid formation and immature bone trabeculae surrounded by large epithelioid osteoblasts with mild pleomorphism (inset). (G) Osteosarcoma (osteoblastic type) demonstrating immature bone formation by cells with morphology ranging from stellate to rounded forms, with basophilic cytoplasm and eccentric nuclei (inset). (H) Osteosarcoma (chondroblastic type) exhibiting mineralized eosinophilic matrix associated with chondroid tissue deposition, along with proliferation of rounded mesenchymal cells with ovoid and pleomorphic nuclei (inset). (I) Osteosarcoma (fibroblastic type) characterized by high cellularity and minimal osteoid matrix. The inset highlights the neoplastic cells showing atypia and hyperchromatism (H&amp;E, 100x; insets, 400x).

Three OTs were identified as OOTs, which frequently exhibited central nidus in the available radiographs (n=2). In addition, some OS cases presented root resorption (n=3), periodontal ligament thickening (n=4) and periosteal reaction (n=4) (Figure 3).

3

Histopathological findings

The kappa coefficient was calculated to assess the interobserver agreement in histopathological analysis, and the agreement between the two examiners was good (=0.72). The histopathological features analyzed in this study are illustrated in Figure 3 and Table 3. Osteomas showed a predominance of the compact subtype, characterized by the deposition of dense lamellar bone, the presence of osteocyte-containing lacunae, minimal medullary tissue, and low cellularity (n=21) (Figure 3A). The cancellous subtype displayed spongy bone with a prominent fibroadipose marrow component (n=17) (Figure 3B). The cellularity was variable but consistently higher than in the compact subtype.

Table 3Osteoid osteomas frequently exhibited features similar to those of conventional OTs, with overlapping characteristics between the compact and cancellous subtypes. In addition, the central nidus was observed at different maturation stages (Figure 3C).

Osteoblastomas frequently exhibited intermediate and high cellularity (n=7 for each category), characterized by marked proliferation of large osteoblasts displaying variable morphologies, predominantly cuboidal and fusiform (n=9). Often immature bone trabeculae were occasionally present alongside areas of osteoid deposition (Figure 3D). Multinucleated giant cells resembling osteoclasts were present in most OBs (n=9) (Figure 3D, detail). Furthermore, two cases were associated with aneurysmal bone cysts (ABCs) (Figure 3E), and no chondroid matrix deposition was observed. A marked presence of atypical epithelioid osteoblasts was identified in three cases (Figure 3F).

Osteosarcomas were predominantly of the osteoblastic subtype and were characterized by proliferative cells of diverse morphology, including rounded, fusiform, or stellate, arranged in solid and dispersed patterns throughout the matrix (Figure 3G). Nonetheless, chondroblastic and fibroblastic OS were also observed in the sample (Figure 3H, I). The neoplastic cells frequently showed indistinct cytoplasmic borders and, occasionally, vesicular nuclei with prominent nucleoli.

Based on the degree of differentiation, OSs were further classified and were mostly well-differentiated (n=12) and exhibited mild atypia (n=11). Cytological alterations included hyperchromatism, cellular and nuclear pleomorphism, increased nucleus-to-cytoplasm ratio, and increased mitotic activity (Figure 3G-I, details).

## Discussion

Bone-forming tumors of the maxillofacial region exhibit variable incidence rates. Due to their clinical-epidemiological complexity, heterogeneity of imaging findings, and their distinctive histopathological features, descriptive studies are important to improve awareness of these lesions to support accurate diagnosis and treatment (5 , 7 - 10). In this context, to the best of our knowledge, this represents one of Brazil's largest retrospective studies of BFTs of the jaws, based on the number of cases analyzed.

Bone-forming tumors accounted for less than 0.5% of the diagnoses reported at the referral service during the study period, reinforcing the low incidence of primary bone tumors in the jaws (16). Osteoma was the most prevalent neoplasm, followed by OB and OS. In addition, the progressive increase in diagnoses observed over time may reflect greater awareness among health professionals regarding the importance of clinical and radiographic information in assisting oral pathologists to establish more accurate final diagnoses. Epidemiological surveys have also highlighted variability in the prevalence of these tumors among different populations (16 - 18). Although infrequent, recent investigations have described particularities and similarities of BFTs in oral and extra-gnathic anatomical locations, reinforcing the importance of the clinicopathological recognition of these lesions for achieving accurate and precise diagnosis (19 - 21).

Benign neoplasms (OT and OB) predominantly affected female patients, consistent with previous observations (5 , 22). In contrast, osteosarcomas showed no gender predilection, in agreement with other reports (9 , 10 , 23 , 24). Although most BFTs were observed in white individuals, it should be emphasized that this is a monocentric study; therefore, the demographic profile may reflect racial disparities in Brazil rather than broader ethnicity-related trends in BFTs.

At the time of diagnosis, patients ranged in age from the first to the seventh decade of life, indicating that BFTs can affect a wide broad age range (3 , 4 , 11 - 13). This study revealed that OB typically presents at a younger age, primarily in the third decade of life, consistent with findings from previous investigations (8 , 22).

Clinically, BFTs predominantly occur in the posterior mandible, as demonstrated in our series (5 , 6 , 22 - 26). While OB and OS frequently presented as swellings, OT typically lacks clinical signs and is therefore detected incidentally on radiographic imaging (7). In this study, most OTs and OBs were asymptomatic and had a clinical duration exceeding six months, further highlighting the indolent nature of these benign neoplasms (7). Additionally, the limited growth of osteoid osteoma tumor (OOT) aids in the differential diagnosis from OB; currently, these entities are arbitrarily distinguished by tumor size, with lesions smaller or larger than 2 cm in diameter being diagnosed as OOT or OB, respectively (3).

It is noteworthy that three OBs in our sample presented rapid progression, symptoms, and sizes exceeding 4 cm, characteristics suggestive of aggressive OB (22 , 26). These observations may also explain the larger mean size of OBs compared to OSs in the present series. Furthermore, OSs were predominantly symptomatic and had a shorter mean duration at diagnosis, reflecting their distinct biological behavior compared to the other groups studied, which is characterized by locally expansive and destructive growth (10). According to our findings, both tumor size and time of evolution were statistically significant, corroborating the trend whereby aggressive tumors, such as OSs, exhibit greater dimensions and more rapid growth.

Although discrepancies in clinical diagnosis were notable, the diagnostic hypotheses generally involved mineralized tissue-producing pathologies, particularly odontogenic tumors and other types of bone diseases, consistent with previous findings (9 , 22). Therefore, despite occasional agreement between clinical and final diagnoses, these results indicate that clinical and radiographic evaluations were important auxiliary diagnostic tools for the clinicians who performed the biopsies.

Radiographic evaluations are essential for detecting lytic or osteogenic processes and serve as fundamental complementary diagnostic tools (27). Benign BFTs predominantly exhibited regular, well-defined, and circumscribed margins (5 , 7 , 18). OTs appeared as radiopaque masses with well-defined sclerotic margins, consistent with their indolent nature (5 , 7 , 26). Cases of OTs that presented a central nidus on radiographic examinations were classified as OOTs (7). Although some authors classify OOTs as a subtype of OB, this study considered them a subtype of OTs, primarily due to their smaller size and the presence of a distinct zone of reactive bone formation (8 , 22). In contrast, OBs typically present with larger dimensions, progressive growth, and lack reactive perilesional bone formation, as intense perilesional sclerosis is observed exclusively in OOTs (8 , 22). Thus, these features may serve as auxiliary diagnostic tools in challenging cases of OTs.

OB and OS predominantly exhibited mixed radiographic patterns and frequently lacked peripheral halos. Some OBs may present atypical radiographic features suggestive of malignancy, particularly in cases with aggressive behavior. Although OBs and OSs can share similar radiographic appearances, OBs, as benign neoplasms, predominantly presented well-defined borders, a feature typically not observed in OSs. These findings reinforce the distinct biological behavior of these neoplasms (8 , 9 , 22 , 24 , 28). All OS cases in this study demonstrated irregular, poorly defined margins and were occasionally associated with aggressive periosteal reactions, root resorption, and widening of the periodontal ligament space, further underscoring their aggressive behavior (1 , 9 , 28).

Histopathological findings observed in BFTs were consistent with previous studies. OTs are primarily of the compact subtype, characterized by abundant lamellar bone with a minimal medullary component. However, some cases may exhibit predominantly spongy bone, fibroadipose marrow, and occasional osteoblastic cells, known as the cancellous subtype (5 - 7). In both patterns, small osteocytes are observed within lacunae (5 - 7).

Analysis of OBs revealed a predominance of moderate to high cellular activity regardless of clinical behavior (aggressive versus non-aggressive). This finding contrasts with Yalcinkaya et al. (8), who reported high cellular density specifically in aggressive cases. Epithelioid osteoblasts, characterized by abundant cytoplasm, hyperchromatic nuclei, and focal clustering, were also observed in clinically aggressive OBs of this study. The lack of differences in cellularity between the two groups may be attributed to the small sample size of OBs in the present series. Furthermore, the coexistence of ABCs in OBs has been reported previously (3 , 8 , 20).

Osteosarcomas were predominantly of the osteoblastic subtype, as observed previously (10 , 24 , 28). Most cases in this study were well-differentiated, in contrast to other reports of OS in the jaws (9 , 24). Variations in the differentiation grade of gnathic OSs may, in part, reflect sample selection bias, as head and neck OSs have previously been described as low- to intermediate-grade neoplasms with better prognosis compared to those at other anatomical sites (28 , 29). Accordingly, histopathological grade has been identified as a significant predictor of survival (23 , 29).

**Figure 4:**
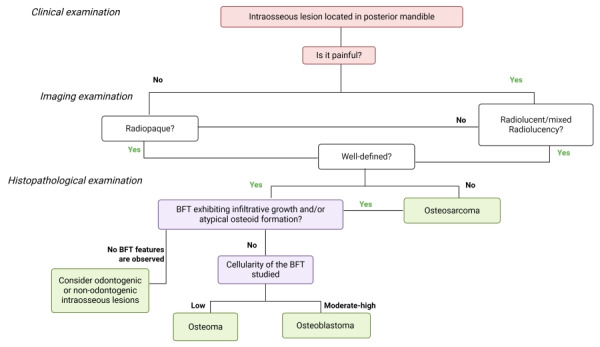
Diagnostic flowchart of bone forming tumors of the jaws.

Therefore, histopathological analysis stands out as a major auxiliary tool for differential diagnosis of BFTs. Whereas OTs present distinctive findings, cases suspected to be OBs must be analyzed with caution (7 , 8 , 22). The absence of a central nidus circumscribed by sclerotic bone renders OOTs histopathologically identical to OBs (8 , 14). The differential diagnosis of clinically aggressive OBs is essential to exclude low-grade osteosarcomas (8). Infiltration of adjacent bone, lack of bone maturation in tumor periphery and presence of sheets of neoplastic cells with no matrix deposition are important features suggestive of OS, whereas the lack of permeative growth into surrounding bone and peripheral maturation favor the diagnosis of OBs (14). In the present study, nuclear atypia, permeative growth, and atypical mitoses were assessed to distinguish these cases, but no such abnormalities were identified. In addition, OBs may occasionally exhibit osteoclast-like, multinucleated giant cells or cartilaginous components (8). However, no chondroid matrix was found, which underscores the rarity of this feature in OBs. A schematic summary of the differential diagnosis of BFTs based on clinical, radiographic, and histopathological features is presented in Figure 4.

4

The strengths of this study include a comprehensive demographic, clinical, radiographic, and histopathological characterization of BFTs in the gnathic bones of a Brazilian population. Additionally, the sample was derived from a major oral diagnostic center in Brazil, thereby making this study a valuable resource for clinicians and oral pathologists seeking to understand the profile of BFTs.

Nonetheless, due to the monocentric, retrospective design, the diagnosed cases originated from a variety of public and private clinics and hospitals. This diverse referral base limits standardization in the completion of biopsy record forms, often resulting in missing data for some cases, which constrained the assessment of certain outcomes. Although our main findings are consistent with previous reports, future multicenter studies are necessary to confirm these trends with greater reliability and validity.

## Conclusions

Our findings confirm that BFTs are relatively uncommon lesions with a predilection for females, most frequently occurring during the fourth decade of life and primarily affecting the posterior mandible. OTs and OBs were the most common BFTs. In contrast to typical OTs and OBs, aggressive OBs and OSs tend to produce apparent clinical signs due to their expansive growth. Radiographically, OTs appear as radiopaque masses, whereas OBs typically exhibit mixed radiolucent-radiopaque patterns. OSs, in turn, present as poorly defined lesions with mixed radiolucent-radiopaque patterns and are often associated with aggressive periosteal reactions, root resorption, and widening of the periodontal ligament space. Prominent histopathological features include the predominance of the compact subtype among OTs and moderate to high cellular activity in OBs, with epithelioid osteoblasts occasionally observed in clinically aggressive cases. Nevertheless, the absence of nuclear atypia, permeative growth, and atypical mitoses distinguishes these lesions from osteoblastic-type OS. The OSs of the jaws are generally low- to intermediate-grade and most frequently exhibit osteoblastic or chondroblastic subtypes.

In summary, BFTs of the maxillomandibular region exhibit clinical, radiographic, and histopathological diversity. By presenting these findings, this study provides new insights that assist dental surgeons in understanding these entities, thereby facilitating accurate diagnosis, appropriate management, and improved prognosis for their patients. Furthermore, these results may inform future investigations into the epidemiology and, ultimately, the pathogenesis of BFTs, thereby contributing to the advancement of knowledge regarding BFTs of the gnathic bones.

## Figures and Tables

**Table 1:** Table Demographic and clinical aspects of bone-forming tumors.

Clinical features	Bone-forming tumors			Total n (%)	p
	OT n (%)	OB n (%)	OS n (%)		
Gendera (n=74)					
Female	32 (78.0)	12 (70.6)	8 (50.0)	52 (70.3)	0.124
Male	9 (22.0)	5 (29.4)	8 (50.0)	22 (29.7)	
Male-to-female (M:F) ratio	1:3.5	1:2.4	1:1.0	1:2.3	
Symptomatologyb (n=55)					
Asymptomatic	26 (83.9)	9 (69.2)	4 (36.4)	39 (70.9)	0.017
Symptomatic	5 (16.1)	4 (30.8)	7 (63.6)	16 (29.1)	
Anatomical locationb (n=72)					
Posterior mandible	27 (67.5)	13 (81.3)	7 (43.8)	47 (65.3)	0.274
Posterior maxilla	0 (0)	0 (0)	2 (12.5)	2 (2.8)	
Anterior maxilla	5 (12.5)	1 (6.3)	1 (6.3)	7 (9.7)	
Anterior mandible	3 (7.5)	1 (6.3)	3 (18.8)	7 (9.7)	
Mandible, NS	2 (5.0)	1 (6.3)	2 (12.5)	5 (6.9)	
Maxilla, NS	3 (7.5)	0 (0)	1 (6.3)	4 (5.6)	
Age (years)c (n=69)					
Mean±SD	40.27±17.8	27.06±14.24	38.18±21.79	36.72±18.66	0.053
Range	7-78	7-55	10-79	7-79	
Duration (months)c (n=47)					
Mean±SD	40.7±34.03	10.8±6.5	5.5±6.93	24.6±29.66	<0.001
Range	5-120	2-24	1-24	1-120	
Size (cm) (n=47)c					
Mean±SD	1.06±0.75	3.25±1.97	2.83±1.42	2.06±1.63	<0.001
Range	0.2-3	0.8-7.0	0.8-5	0.2-7.0	
Clinical Diagnosisb (n=74)					
Concordant	24 (58.5)	4 (23.5)	6 (37.5)	34 (45.9)	0.041
Discordant	17 (41.5)	13 (76.5)	10 (62.5)	40 (54.1)	

aPearson’s chi-square test; bFisher’s exact test; cKruskal-Wallis Test. NS: Not Specified.

**Table 2:** Table Radiographic characteristics of bone-forming tumors.

Radiographic features	Bone-forming tumors			Total n (%)	pa
	OT n (%)	OB n (%)	OS n (%)		
Radiodensity (n=68)					
Mixed radiolucency	8 (21.1)	12 (75)	7 (50)	27 (39.7)	<0.001
Radiopaque	26 (68.4)	3 (18.8)	2 (14.3)	31 (45.6)	
Radiolucent	4 (21.1)	1 (6.3)	5 (35.7)	10 (14.7)	
Border of the lesion (n=33)					
Well-defined	13 (81.3)	6 (66.7)	0 (0)	19 (57.5)	<0.001
Poorly defined	3 (18.8)	3 (33.3)	8 (100)	14 (42.5)	
Halo (n=32)					
Radiolucent	4 (22.2)	0 (0.0)	0 (0.0)	4 (12.5)	0.268
Radiopaque	8 (44.4)	3 (33.3)	1 (20.0)	12 (37.5)	
Absent	6 (33.3)	6 (66.7)	4 (80.0)	16 (50.0)	

aFisher’s exact test.

**Table 3:** Table Absolute and relative distribution of bone-forming tumors according to the histopathological parameters.

Bone-forming tumors	Subtype
	n (%)
Osteoma	
Compact	21 (51.2)
Cancellous	17 (41.5)
Osteoid	3 (7.3)
Osteoblastoma	17 (100.0)
Osteosarcoma	
Osteoblastic	9 (56.3)
Chondroblastic	5 (31.3)
Fibroblastic	2 (12.5)

3

## Data Availability

Declared none.
